# Virtual reality in neurologic rehabilitation of spatial disorientation

**DOI:** 10.1186/1743-0003-10-17

**Published:** 2013-02-08

**Authors:** Silvia Erika Kober, Guilherme Wood, Daniela Hofer, Walter Kreuzig, Manfred Kiefer, Christa Neuper

**Affiliations:** 1 Department of Psychology, University of Graz, Universitaetsplatz 2/III, 8010, Graz, Austria; 2 Privatclinic Lassnitzhoehe, Lassnitzhoehe, Austria; 3 Laboratory of Brain-Computer Interfaces, Institute for Knowledge Discovery, Graz University of Technology, Graz, Austria

**Keywords:** Topographical disorientation, Brain damage, Way-finding training, Virtual rehabilitation, Visuo-spatial memory, Visual navigation

## Abstract

**Background:**

Topographical disorientation (TD) is a severe and persistent impairment of spatial orientation and navigation in familiar as well as new environments and a common consequence of brain damage. Virtual reality (VR) provides a new tool for the assessment and rehabilitation of TD. In VR training programs different degrees of active motor control over navigation may be implemented (i.e. more passive spatial navigation vs. more active). Increasing demands of active motor control may overload those visuo-spatial resources necessary for learning spatial orientation and navigation. In the present study we used a VR-based verbally-guided passive navigation training program to improve general spatial abilities in neurologic patients with spatial disorientation.

**Methods:**

Eleven neurologic patients with focal brain lesions, which showed deficits in spatial orientation, as well as 11 neurologic healthy controls performed a route finding training in a virtual environment. Participants learned and recalled different routes for navigation in a virtual city over five training sessions. Before and after VR training, general spatial abilities were assessed with standardized neuropsychological tests.

**Results:**

Route finding ability in the VR task increased over the five training sessions. Moreover, both groups improved different aspects of spatial abilities after VR training in comparison to the spatial performance before VR training.

**Conclusions:**

Verbally-guided passive navigation training in VR enhances general spatial cognition in neurologic patients with spatial disorientation as well as in healthy controls and can therefore be useful in the rehabilitation of spatial deficits associated with TD.

## Introduction

Impaired spatial orientation is a common consequence of brain damage that greatly reduces the quality of life and autonomy in daily living of neurologic patients. To date, no standard rehabilitation of spatial abilities after brain damage is in use [1]. One frequent form of spatial disorientation is the topographical disorientation (TD) [2,3].

Virtual reality (VR) technique offers the opportunity to create complex individualized and natural simulated environments, in which specific spatial deficits, such as egocentric disorientation or the ability to recognize landmarks, can be assessed precisely and in an ecologically valid way [4-13]. In comparison to more traditional assessment methods such as paper-and-pencil measures, VR offers the tools for simulating realistic spatial navigation under controlled experimental conditions.

Beside the use of VR as an assessment tool for spatial deficits, there are also few studies using VR for training spatial abilities in patients with orientation problems [5,14,15]. Virtual training environments offer the possibility to train specific spatial deficits associated with TD, such as egocentric or allocentric (e.g. landmark agnosia) orientation problems [2,7-9]. One of the first studies using VR in rehabilitation of navigational skills was a single-case study by Brooks et al. (1999). A patient with amnesia who showed memory and orientation deficits was trained in route finding around a real and a virtual version of the same hospital unit [16]. Following the VR training, the patient was able to successfully perform routes in the real unit. Hence, the patient easily transferred the learned performance from the virtual to the real world [17]. Astonishingly, the same patient failed to learn the routes in the real unit. Rose et al. (2001) extended the findings by Brooks et al. (1999) and trained four more patients with amnesia on route learning in VR. The virtual training was as successful as the real world training [18]. Moreover, Wilson et al. (1996) successfully trained physically disabled children in a virtual building with the goal of finding the fire extinguishers and fire door locations in the real building [19]. Caglio et al. (2012) used a 3D video game as navigational training program, which led to improvements in spatial memory in a brain damaged patient [15].

Brooks et al. (1999) attributed the superiority of VR training over real world learning to three different reasons: First, the routes can be performed more often in the virtual than in the real world, since the routes can be performed faster in VR. Second, in VR the patients are not restricted by any physical disabilities. Different degrees of active motor control over navigation may be implemented depending on individual motor coordination abilities. Navigation can be self-paced by means of a joystick [16], a keyboard [20], or verbal instructions [21], or passive [22], when participants exert no control over navigation. Third, in VR there are no unexpected distractions that could interrupt the patients during learning as in the real world.

In the present study, we have designed a VR-based verbally-guided passive navigation training program, which should be suitable for neurologic patients with spatial disorientation: The first aspect of our training program is to achieve positive results in a short period of time. Brooks et al. (1999) and Rose et al. (2001) found positive training effects after three and two weeks of daily training, respectively [16,18]. For the sake of economy, the route finding training was reduced to five training sessions of 20 min each in the present study. Hence, we assumed that five training sessions in VR are sufficient to improve spatial orientation in neurologic patients. The second aspect refers to the degree of motor control necessary for performing training. Rose, Brooks, Attree, et al. (1999) compared the influence of active (active navigation through the VR, controlled movement through the VR) and passive (passively watching, did not control movement through the VR) navigation through a virtual building on the development of spatial knowledge with vascular brain injury patients and control participants. They demonstrated a superiority of active over passive navigation [23]. However, for many patients active navigation may lead to cognitive overload since controlling motor performance requires memory and mental flexibility resources [24], which are also necessary for route learning. Furthermore, positive effects of navigation training have been reported for passive navigation as well [5,22,23]. This is especially important because not every patient is able to move a joystick or use a keyboard to control navigation. For this reason we decided to implement a verbally-guided passive navigation training, in which navigation was controlled by verbal commands given by participants to the experimenter. A pilot study in healthy elderly participants indicated that this approach is reasonable. The third aspect refers to the learning mode employed in training programs. Lloyd et al. (2009) could show that patients with brain injury benefit more from errorless learning in a virtual route learning task compared to trial and error learning [21]. For instance, Brooks et al. (1999) corrected the patient who underwent a spatial training in their study, whenever the patient took a wrong turn as well [16]. In the present study, patients were instructed to correct their route by means of verbal feedback from the experimenter whenever they took a wrong route during navigating in virtual scenarios. Thereby one can avoid patients “getting completely lost” in virtual environments and may accelerate the learning process. The fourth aspect of the present route finding training was to design a standardized virtual environment which is unfamiliar to all participants but at the same time is realistic enough to provide a rich learning environment for participants. The training of participants in unfamiliar environments controls for the effects of previous experience and habits on navigation performance [25]. Furthermore, it increases the comparability of training outcomes across participants and participants’ groups and also improves the accuracy of performance assessment.

A final question of the present study is whether the obtained training outcomes can be generalized to broader aspects of spatial cognition. Are there only learning effects, which are specific to trained routes and training materials or can one observe more general cognitive changes in trained patients? Is it possible to track these changes using well established psychometrically validated neuropsychological instruments? Results reported by Brooks et al. (1999), Rose et al. (2001), Wilson et al. (1996), and Lloyd et al. (2009) already present high ecological validity and demonstrate the utility of VR-based training programs for improving spatial navigation. However, these studies did not examine how more general aspects of spatial cognition may have been changed by spatial training. In this context, Durlach et al. (2000) mentioned that prior VR studies only trained specific spatial behaviors in specific virtual or real spaces, and that there is a lack of VR studies that trained spatial behavior in general, such as improving a participant’s general spatial abilities and skills. The aim of VR-based spatial rehabilitation should be to train improved spatial performance of a variety of types in different spaces [14]. Therefore, we addressed the question, whether spatial training in a specific virtual space can enhance general spatial abilities assessed with standardized neuropsychological tests in neurologic patients with spatial disorientation as well as in a healthy matched control group.

In summary, the present study pursues two main goals. The first is to demonstrate the proof-of-principle of the applied VR-based verbally-guided passive navigation training program in neurologic patients with spatial orientation problems and healthy participants. We expect that both patients and controls will benefit from VR-based verbally-guided passive navigation training, because it combines training parameters (verbally-guided passive navigation, errorless learning mode, standardized unfamiliar virtual environment, duration of training) that led to performance benefits in both populations. The second aim is to address the question of how large is the transference of improvements in VR route training to more general aspects of spatial cognition and how consistent are these transference effects in and across populations. Prior studies showed that navigation training in a virtual environment can improve visual-spatial learning in neurologic patients [15]. Exploration of a new and complex environment, such as the virtual city in the present VR navigation training program, recruits spatial memory resources (e.g. short-term spatial memory, spatial learning rates). When the participants need to remember a route, the demands on spatial memory resources are particularly high. Moreover, when the participants need to navigate on a route from a start point to an endpoint and vice-versa, participants have to execute a series of mental transformations on the representation of the route. This transformation process during the VR navigation training is related to the ability to imagine spatial objects and to mentally transform them. Finally, the more realistic the VR model, the higher the demands on visual orientation performance necessary to filtrate useful input from visual noise from the background. For these reasons, we expect that positive training effects achieved with VR-based verbally-guided passive navigation training will potentially induce positive changes in measures of spatial memory, mental transformations and visual orientation performance.

## Methods

### Participants

Eleven neurologic patients (5 men, 6 women) that showed severe impairments in spatial orientation performance in their ordinary environments (e.g. hospital area, home) were recruited among the inpatients of the Neurology Unit of the Privatclinic Lassnitzhoehe, Austria. Ten patients had lesions in the right hemisphere, one patient had lesions in the left hemisphere. The most common etiology of the neurologic disorders was stroke. A detailed description of the patient group is shown in Table 1. Patients were assigned to the study based on the diagnostics of spatial orientation disorders made by their attending doctors [6,9]. The neurologic healthy control group (5 men, 6 women) was recruited from the Orthopedic Unit of the same clinic (matching criteria: sex and age). Mean age of the patient group was 66.09 (*SE* = 3.30) years and of the matched control group 66.18 (*SE* = 2.97) years. Participants who scored less than 17 points on the Mini-Mental State Examination [8] and patients with visuo-spatial hemineglect, severe language impairments, major psychiatric illness and depression were excluded from the study. All participants included in the study had normal or corrected-to-normal vision. The study conforms with the code of ethics of the World Medical Association (WMA, Declaration of Helsinki) [26].

**Table 1:** Patient description

**Patient code**	**Age (years)**	**Sex**	**Diagnosis**	**Affected hemisphere**	**Lesion location**	**TSO§**	**Further information**
1	73	male	stroke	right	arteria cerebri media	5	
2	79	female	stroke	right	arteria cerebri media	6	Moderate memory and attention deficits (assessed by the SKT – Syndromkurztest zur Erfassung von Gedächtnis- und Konzentrationsstörungen)
5	75	male	stroke	right	fronto-parietal	9	Subdural hematoma, marginal symptoms of dementia
6	80	female	stroke	right	arteria cerebri media, basal ganglia	5	Left-sided hemiparesis
8	58	female	aneurysm and subsequent infarcts	right	arteria cerebri posterior	170	Subarachnoid hemorrhage: HUNT and HESS II, Quadrantanopia
10	59	male	stroke	right	arteria cerebri media, thalamus, basal ganglia	14	Left-sided hemiparesis
11	57	male	stroke	right	basal ganglia	14	Left-sided hemiparesis
12	72	male	stroke	right	arteria cerebri media	5	
14	68	female	cerebral haemorrhage	right	arteria cerebri media	6	Diplopia, headache
17	61	female	aneurysm and subsequent infarcts	right	arteria communicans anterior, parietal infarct	30	Organic brain syndrome, moderate memory and attention deficits (assessed by the SKT)
18	45	female	traumatic brain injury	left	hippocampus, pons	12	Attention deficits (assessed by Cognitrone), memory deficits (assessed by Wechsler Memory Scale)

*§TSO*: Time since onset (weeks).

### Apparatus and materials

In order to assess participants’ general spatial abilities, standardized neuropsychological tests were used. Participants were asked to complete four spatial tests before and after the five VR training sessions: the Benton Test, the LPS 50+, the LVT, and the CBTT. In pre-and post-test measures parallel forms of the Benton Test and the LPS 50+ were used. All tests were conducted on one day in the pre- and post-measurement, respectively. The overall duration of the pre- and post-measurement was about 45 min each (including written informed consent, assessment of demographic and basic stroke-related data, instructions, Benton Test, LPS 50+, LVT, and CBTT).

The Benton Test, which is also called Benton Visual Retention Test, assesses visual perception and visual memory [27]. It is also used in clinical diagnosis of brain damage. The participant is shown for ten seconds 15 standardized cards with geometric forms, one at a time. Then, the participant is asked to recognize the previously shown card under four different cards (multiple-choice form). Two parallel forms of the multiple-choice form of the Benton test are available. The highest score one can reach is 15 points. Participants older than 55 years get an extra point. A score of 13 points is associated with a normal visual perception and visual memory performance. Scores lower than 12 points are indicators of impaired visual perception and memory [27]. For the multiple-choice form, a moderate internal consistency is reported, with a split-half reliability of about 0.76. The Benton Test assessing visual-spatial memory was used to assess possible improvements in visual-spatial learning due to the VR navigation training.

The Achievement Measure System 50+ (Leistungspruefsystem 50+, LPS 50+) is a German standardized intelligence test developed for older people between 50 and 90 years. It is based on different subtests, which are designed to measure Thurstone’s Primary Mental Abilities [28]. The LPS 50+ includes seven subtests assessing verbal knowledge, non-verbal reasoning, verbal fluency, spatial imagination, flexibility of closure and verbal closure. For the present study, only one subtest assessing spatial imagination was used. In this subtest, the participant is shown different geometric objects. The participant is asked to count the number of surface areas of these objects. For this test two parallel versions are available. The spatial imagination subscale shows good values in reliability, with a split-half reliability of 0.96 and a test-retest reliability of 0.94 [29]. The LPS 50+ was used to assess possible improvements in the ability to imagine spatial objects and to mentally transform them due to the VR navigation training.

The Visual Pursuit Test (Linienverfolgungstest, LVT) [30] is a standardized computer based subtest of the Vienna Test System [31]. It measures the visual orientation performance for simple structures in a complex environment. The participant is required to work in a focused way, to ignore distractions, while being placed under time pressure. Hence, this test is also suited to assess selective visual attention. The initial practice phase combines the instruction and eight practice items. In the subsequent test phase, the participant is presented with an array of lines and must find the end of a specified line as quickly as possible within a given time. For the present study, the screening form of the LVT was used containing 18 items. For statistical analyses, the total score of the LVT and the median time of correct answers (sec) were used. The LVT is an internally consistent measure, with a Cronbach’s alpha coefficient of 0.92 [30,31]. We used the LVT to assess possible changes in the visual orientation performance for simple structures in complex environments due to the VR training.

The Corsi Block-Tapping Test (CBTT) [32] is a standardized computer based subtest of the Vienna Test System [31]. It assesses the so called “immediate block span”, which is associated with visual short-term memory capacity and implicit visuo-spatial learning. The participant views nine irregularly positioned blocks on a screen and a pointer taps on a number of these blocks in turn. Afterwards, the participant is required to tap with a special pencil on the same blocks in the same order. The number of blocks increases by one after three items. When the participant makes an error in three successive items the test stops. The CBTT shows good values in reliability between 0.81 and 0.89 [31]. The CBTT assessing visual short-term memory was used to assess possible improvements in visual-spatial learning due to the VR navigation training. Due to motor impairments, not all patients suffering from brain damage were able to complete the computer based tests LVT and CBTT. Therefore, only seven patients with spatial orientation disorders completed the LVT, and eight patients with spatial deficits completed the CBTT.

**Figure 1:**
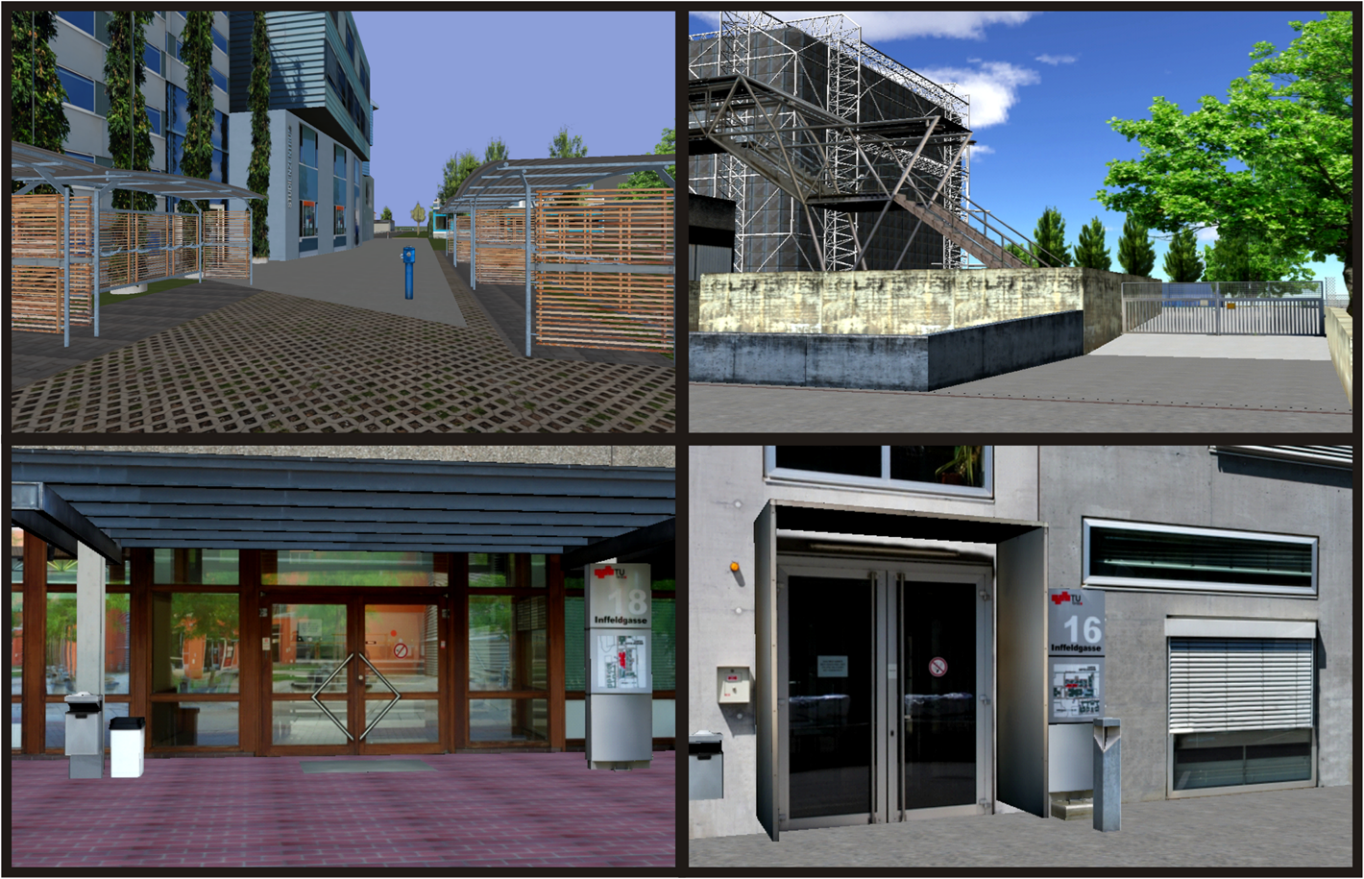
Sample views of the virtual environment used for the VR-based spatial navigation training program, which was a district of the real world town of Graz, Austria.

The virtual environment was a simulation of a district of the real world town of Graz, Austria (see Figure 1). The virtual environment was generated by the Institute of Computer Graphics and Knowledge Visualization (CGV) of the Graz University of Technology (http://www.cgv.tugraz.at). The virtual 3D model of the district of Graz was generated by using aerial and first-person view photos of the real world unit with the framework instantreality (http://www.instantreality.org). None of the participants had been in the corresponding real world district of Graz before. The virtual city was presented on a 2x2 m projection screen via a conventional projector in a monoscopic view. During the VR training, different routes were presented, which the participants had to learn and recall correctly. Each route contained three decision points (left/right turns or straight-ahead choices). Navigation speed and direction in VR were controlled by joystick. In a pilot study, the ability of elderly participants to control the joystick by themselves was assessed. Pilot testing showed that it was too difficult for elderly to operate the navigation joystick and simultaneously to concentrate on the routes. Furthermore, due to motor impairments, some patients were physically unable to efficiently control the joystick. For these reasons, in the present study a verbally-guided passive navigation training program was adopted. In this training program participants gave oral commands such as “straight ahead”, “turn left”, “turn right” and “stop” to the experimenter, who was in charge of operating the joystick. Therefore, participants controlled navigation indirectly by means of verbal commands and not actively by means of motor responses.

VR training program: The VR training was a route finding training. Participants were shown one route, completely directed by the experimenter in the learning phase. The experimenter pointed out each junction of the route and stated the action to be taken before executing it. Hence, the experimenter gave verbal instructions such as: “We are approaching a crossroad now. We have to turn left here.” In the subsequent retrieval phase, participants had to call out the correct directions to the experimenter at each junction. If the participant took a wrong turn, the experimenter informed the participant and returned to the correct route in order to assure errorless learning [21]. In the present study, learning and retrieval of one route were repeated until the participant made no mistake in the retrieval phase. One training session took approximately 20 min in which maximal three different routes could be learned. In each of the five training sessions different routes were presented. The performance in the VR training task was quantified by calculating a weighted total score (see Appendix I for further details), including the number of mistakes per route and the number of correctly learned routes per training session. Higher total scores are associated with better performance in the VR retrieval task.

### Procedure

The five VR training sessions and the pre- and post-assessment of general spatial abilities using standardized neuropsychological tests took place in the rehabilitation clinic. In the first session, participants gave written informed consent. Demographic and basic stroke-related data were also collected. Afterwards, general spatial abilities were assessed using the Benton Test, LPS 50+, LVT, and CBTT. In the sessions 2–6, the five VR training sessions were conducted. In each 20-min training session, participants had to learn and recall up to maximal three different routes. Before and after each training session, participants filled out the Simulator Sickness Questionnaire (SSQ) [33]. The SSQ was developed to determine whether users of virtual environments experience cybersickness symptoms, which can confound the data. The analysis of the SSQ revealed that the participants showed no sickness symptoms during the five VR training sessions. Additionally, patients and controls showed no differences in their SSQ-ratings (all *p* > 0.05). In the seventh session, general spatial abilities were assessed again by using the Benton Test, LPS 50+, LVT, and CBTT. In pre-and post-test measures parallel forms of the Benton Test and the LPS 50+ were used to avoid learning effects.

## Results

### ANOVAs

#### Training data

The training data were assessed using repeated-measures ANOVA models in which time (training sessions 1, 2, 3, 4, and 5) was defined as a 5-levels within-subjects factor and group (patients × matched controls) as a between subjects factor. The training data revealed a significant main effect of time (*F*(4, 80) = 3.08; *MSE* = 398; *p* = .02), which was complemented by a linear contrast (*F*(1,20) = 8.12; *MSE* = 1561; *p* = .01) and a main-effect of group (*F*(1, 20) = 321; *MSE* = 206549; *p* < .001). Together these results indicate that matched controls showed superior performance in VR training than patients regardless of the time point. Moreover, both controls and patients benefited from VR training, as the performance of both increased with training in a linear fashion.

#### Pre-post-assessment

The performance in general spatial abilities was assessed in the pre- and post-tests using repeated-measures ANOVA models in which time (pre-test × post-test) was defined as a within-subjects factor and group (patients × matched controls) as a between subjects factor. Regarding performance in the LPS 50+, significant main effects of time (*F*(1, 20) = 12.64; *MSE* = 134.75; *p* = .002) and group (*F*(1, 20) = 1160; *MSE* = 124339; *p* < .001) were observed but a non-significant interaction time × group (*F*(1, 20) = 3.24; *MSE* = 34.57; *p* = .087). Similar results were observed in the Benton Test: Significant main effects of time (*F*(1, 20) = 31.03; *MSE* = 76.46; *p* < .001) and group (*F*(1, 20) = 1522; *MSE* = 7127; *p* < .001) were observed but a non-significant interaction time × group (*F*(1, 20) = 1.32; *MSE* = 3.27; *p* = .263). In contrast, in the total score of the LVT no main-effect of time (*F*(1,20) = 2.81; *MSE* = 1.84; *p* = .11) or interaction time × group (*F*(1,20) <1 n.s.) were observed, but only a main effect of group (*F*(1,20) = 178; *MSE* = 49178; *p* < .001). In the median time score, significant main effects of time (*F*(1,20) = 9.88; *MSE* = 4.81; *p* = .005) and group (*F*(1,20) = 35.74; *MSE* = 997; *p* < .001) were observed as well as the interaction time × group (*F*(1,20) = 5.41; *MSE* = 2.64; *p* = .031). Finally, considering the scores of the CBTT, only a main effect of group was observed (*F*(1,20) = 159; *MSE* = 918; *p* < .001).

The main effect of group indicates that the controls performed better than the patients at all occasions. Therefore, the subsequent t-tests were calculated for the patient and control group separately. Importantly, the main effect of time indicates that both, controls and patients improved their performance in measures of general spatial abilities after VR training. Finally, the significant interaction time × group observed in the median time scores of the LVT indicates that patients benefited more from VR training than matched controls.

### T-Tests

#### Training data

**Figure 2:**
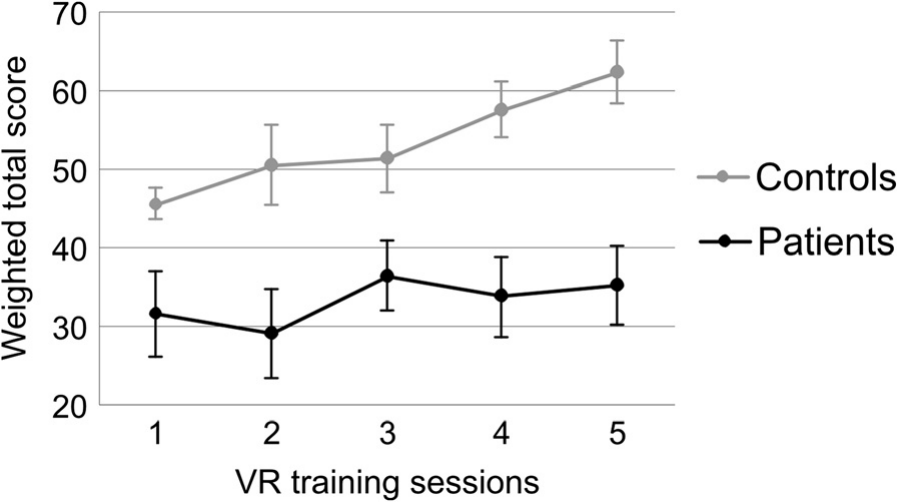
Means and standard errors of the route finding performance (weighted total score including the number of mistakes per route and the number of correctly learned routes per training session) in the VR way-finding training for the five training sessions, separately for each group.

To assess improvements in VR route finding ability, weighted total scores obtained in the first and the fifth training sessions were compared for the patient and control groups separately (Figure 2). Because of the multiple comparisons problem we reduced the number of calculated t-tests by comparing only the first and the fifth session. Performance in the five VR training sessions is illustrated in Figure 2 and presented separately for each group. In the control group, the route finding ability in the VR task improved significantly from the first to the fifth training session (*p* < 0.01) (see Figure 2, Table 2). In the patient group, only a small trend towards an improvement in route finding performance was observed over the five training sessions. Means and standard errors of the behavioural data and the results of the statistical analyses (t-tests) are summarized in Table 2.

**Table 2:** Means and standard errors of the behavioural data and the results of the statistical analyses (t-tests)

	**Patient group**			**Control group**		
	**Pre-test**	**Post-test**		**Pre-test**	**Post-test**	
	***Mean (SE)***		***t-value (df)***	***Mean (SE)***		***t-value (df)***
LPS 50+ score [T-score]	44.73 (1.91)	50.00 (2.04)	−3.32** (10)	58.09 (2.71)	59.82 (2.51)	−1.49 (10)
Benton Test score [Raw-score]	10.18 (0.88)	13.36 (0.43)	−3.79** (10)	12.64 (0.45)	14.73 (0.36)	−4.80** (10)
LVT total score [T-score]	28.43 (1.43)	31.29 (2.77)	−0.93 (6)	46.36 (2.18)	49.36 (1.94)	−1.65 (10)
LVT median time [s]	9.17 (2.08)	7.36 (1.63)	3.28* (6)	4.35 (0.16)	4.18 (0.16)	1.27 (10)
CBTT [Raw-score]	4.38 (0.38)	4.63 (0.26)	−1.00 (7)	5.55 (0.21)	6.18 (0.46)	−1.41 (10)
VR route finding performance [weighted total score] in first (pre-test) and fifth (post-test) VR training session	31.54 (5.44)	35.30 (5.02)	−0.73 (10)	45.36 (1.76)	62.39 (4.03)	−3.59** (10)

Significant results are marked with asterisks (*p < 0.05, **p < 0.01).

The number of participants showing increased, constant or decreased performance in the VR training between the first and last training sessions was determined separately in the two groups. A significant chi-square *Χ*^*2*^(2) = 23.38, *p* < 0.01 revealed that more control participants than patients benefited from route finding training (Table 3). Nevertheless, a substantial proportion of the patient group (45%) still showed improvement in route finding after training (Table 3).

**Table 3:** VR training outcome in patients and controls

**Group**	**Increase**	**Constant**	**Decrease**	***N***
Patients	5	4	2	11
	45%	36%	18%	100%
Controls	7	2	2	11
	64%	18%	18%	100%

Number and percentage of participants showing either an increased, constant or decreased VR navigation performance in the fifth compared to the first VR training session.

#### Pre-post-assessment

**Figure 3:**
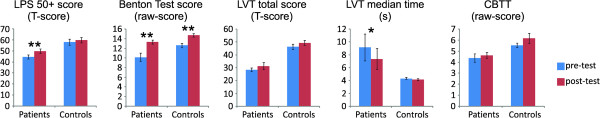
**Bar graphs show means and standard errors of the behavioral data separately for the patient and control group and the results of the statistical analyses (t-tests: pre- vs. post-test).** Significant results are marked with asterisks (*p < 0.05, **p < 0.01).

To complement the investigation on VR training related changes in general spatial abilities, performance in neuropsychological tests was compared between pre- and post-test separately for the patient and control group using t-tests (Figure 3). The patient group showed a significant higher LPS 50+ score in the post-test compared to the pre-test (Figure 3, Table 2). Additionally, after VR training the patient group answered faster in the LVT than before VR training (Figure 3). In both groups, the performance in the Benton Test was higher in the post-test than in the pre-test (Figure 3).

### Single-case analyses

Single-case analyses based on the approach defined by Huber [34] were conducted separately for each individual. This analysis identifies those individuals presenting a positive difference in performance in post-test when compared to the pre-test, which is superior to a critical difference. This critical difference describes the difference in performance which, on the one side, cannot be attributed to random performance fluctuations and, on the other side, occurs rarely in the population. The critical difference is calculated for psychometrically well constructed instruments and useful only for tests with moderate or high reliability. In the present study, critical differences were calculated for the tests LPS 50+, Benton Test, LVT total score. No critical difference could be calculated for the LVT median time and CBTT because of poor or non-existent norms. As shown in Table 4, patients reached critical differences eight times, while controls reached critical differences five times. A critical difference is considered significant when the difference between pre- and post-test shown by the single participants is larger than the critical difference which can be detected by each test (error probability α < 5%) and only occurs in the population with a probability lower than α < 10%.

**Table 4:** Results of the single-case analyses

**UPN**	**Group (1=patient)**	**LPS 50+**	**Benton Test**	**LVT – total score**	LVT – median time *	CBTT *
1	1	+§	+	+§	+	=
2	1	+	+§			
5	1	+	+	=	=	+
6	1	+	+§			
8	1	+	+	=	+	+
10	1	+	+			
11	1	+§	-			-
12	1	+	+§	=	+	=
14	1	-	+	-	+	=
17	1	+§	+	=	+	+
18	1	+§	+	+	+	=
3	2	+	+	+	+	=
4	2	=	=	+	-	=
7	2	+	+	+	+	+
9	2	+	+	+§	+	=
13	2	-	=	+	+	=
15	2	=	+	-	+	+
16	2	+	+	+§	+	=
19	2	+§	+	-	=	=
20	2	-	+§	+§	+	-
21	2	+	=	-	-	+
22	2	+	+	+	+	+

§ Individuals showing a significant improvement in performance in single-case analyses (Huber, 1973). * Because of the lack of normed values for these tasks, the single-case analysis could not be performed.

In these analyses the difference in performance (pre- vs. post-test) obtained by each individual is compared to critical values obtained from published norms. (+) Performance was higher in post- compared to pre-test; (−) Performance was lower in post- compared to pre-test; (=) Performance was in pre- and post-test the same.

### Correlation between VR performance and general spatial abilities

To investigate whether the VR navigation task is a valid assessment method of spatial skills we examined if there is a relationship between route finding ability in the VR task and general spatial abilities assessed before and after VR training [13,35]. For all participants, the weighted total score of VR performance in the first training session was correlated separately with the results of all neuropsychological tests (Benton Test, LPS 50+, total score and median time of correct answers of LVT, CBTT) assessed during the pre-test (Table 5). An increased performance in the VR route finding task was associated with an enhanced performance in all neuropsychological tests. Additionally, the weighted total score of the VR performance in the last VR training session was correlated separately with the results of the neuropsychological tests (Benton Test, LPS 50+, total score and median time of correct answers of LVT, CBTT) assessed during the post-test (Table 5). As in the pre-test, higher scores in general spatial ability tests were associated with an enhanced VR performance (except for the correlation between VR performance and the LPS 50+ score, which showed no association). Statistical comparisons between correlations revealed no changes in the strength of the association between neuropsychological tests and training performance as measured in the pre- and post-tests (Table 5).

**Table 5:** Pearson’s correlations between the weighted total score of the VR performance in the first and last training sessions and results of the neuropsychological tests (Benton Test, LPS 50+, total score and median time of correct answers of LVT, CBTT) during the pre- and post-tests averaged over participants

	**Benton Test ( *N *=22)**	**LPS 50+ ( *N *=22)**	**LVT – total score ( *N *=18)**	**LVT – median time ( *N *=18)**	**CBTT ( *N *=19)**
Total score of VR performance in 1st training session	0.56**	0.43*	0.55*	−0.78**	0.71**
Total score of VR performance in 5th training session	0.51*	0.26 *n.s.*	0.56*	−0.57*	0.49*
Comparison of pre- and post-test correlations with respective training sessions (z-test)	0.22 *n.s.*	0.60 *n.s.*	−0.04 *n.s.*	−1.09 *n.s.*	0.99 *n.s.*

*n.s.* non-significant, *p < 0.05, **p < 0.01.

## Discussion

In the present study we used a VR-based route finding training to improve general spatial abilities in neurologic patients with spatial disorientation and healthy controls. The route finding ability in the VR task increased linearly over the five VR training sessions in both, patients and controls. After the VR training, patients as well as controls improved their general spatial abilities in comparison to the spatial performance before the VR training, assessed with standardized neuropsychological tests. Additionally, performance in the VR route finding task was positively correlated with performance in the standardized neuropsychological tests. In the following paragraphs, these results are discussed in more detail.

### Proof-of-principle of the applied VR-based verbally-guided passive navigation training program

In the present study, we designed a VR-based verbally-guided passive navigation training program. Our results provide evidence that this training program is suitable for neurologic patients with spatial disorientation as well as neurologic healthy controls. The neurologic patient group would not have been able to actively control the navigation joystick adequately because some patients had motor impairments. The errorless learning mode assured that participants could concentrate on the correct routes in VR and that the spatial learning process was not disturbed by taking any unplanned detours. The standardized virtual environment was unfamiliar to all participants as well. In summary, the verbally-guided passive navigation mode, the errorless-learning strategy, and the use of an unfamiliar and realistic standardized virtual environment for the VR route finding training were suitable for both, neurologic patients with spatial orientation problems and healthy controls. This short VR training led to an improvement in spatial navigation performance in VR as well as to a performance improvement in general spatial abilities.

The performance in the virtual route finding task increased in a linear fashion over the five VR training sessions in both, healthy controls and neurologic patients, as indicated by the results of the ANOVA analyses. As expected, healthy elderly with no acquired brain injuries showed a better performance in recalling different routes in VR correctly than the patient group in all training sessions. Hence, even healthy elderly with no spatial deficits can benefit from spatial training in VR and learn to improve their navigational performance due to the used VR way-finding training paradigm. The performance of the patient group also slightly increased from the first to the last session, from an average score of 31.54 points in the first session to 35.30 points in the last session. Furthermore, the patients with spatial disorientation became gradually more confident and also reported to have fun during the spatial training across five VR sessions. Therefore, the initial fear of the new technology and the associated technology gap, which was most prominent in the patient group, disappeared after a few training sessions. Morganti (2004) reported such a technology gap in older participants, too. Generally, the elderly are not familiar with VR interfaces and particular devices [36]. But Morganti (2004) also mentioned that older participants show a clear enthusiasm in embracing such type of rehabilitation when they are “forced” to use VR devices. In this context, it seems reasonable to assume that older participants who suffer from a brain injury, such as the patient group in the current study, show an even more pronounced technology gap than healthy old people. Hence, older people with brain damage probably need more VR training sessions to familiarize themselves with the VR technology than the healthy controls. Brooks et al. (1999) and Rose et al. (2001) carried out much more than five VR training sessions to increase spatial performance in amnestic patients [16,18]. Additionally, 45% of the neurologic patients could show an increased route finding performance in the VR task after only five VR training sessions. This result leads to the conclusion that positive training effects can be observed in neurologic patients as well as healthy participants after five short VR training sessions in route finding.

However, the comparability of the present study with the results of prior VR training studies such as the study of Brooks et al. (1999) or Rose et al. (2001) is restricted due to different VR systems used. For instance, in the study of Brooks et al. (1999) the 3D non-immersive virtual environment was run on a conventional computer screen. Hence, the virtual environment was presented in a monoscopic view on a small screen. In the present study, the VR was presented in a monoscopic view too, but on a large projection wall (2×2 m). There is evidence that technological factors such as screen size can influence VR experiences, e.g. the level of immersion [37]. It is a matter of further research to investigate the effects of technological VR factors on the outcome of VR-based rehabilitation programs. Furthermore, the trained neurologic patient sample in the present study is not comparable with the amnestic patients in the studies of Brooks et al. (1999) and Rose et al. (2001). This restricts the comparability of the studies too.

### Generalization of VR training to general spatial cognition

The second main research question addressed the transference of improvements in VR route training to more general aspects of spatial cognition and the homogeneity of these transference effects across participants. Neurologic patients with spatial orientation deficits as well as neurologic healthy controls were evaluated for different aspects of spatial cognition before and after performing a VR-based spatial navigation training program.

The patient group showed an increased performance in three out of four neuropsychological tests in the post- compared to the pre-test, whereas the matched control group showed a performance improvement in only one test. Patients showed a significant higher LPS 50+ score, a higher score in the Benton Test and faster reaction times in the LVT after VR training compared to the pre-test. Controls showed a performance improvement in the Benton Test. Hence, these results indicate that spatial training in VR can increase general spatial abilities and that VR training seems to be most beneficial for neurologic patients with spatial deficits. The results of the single-case analysis support this finding. Altogether, neurologic patients reached critical differences eight times (seven out of eleven patients), which means that the performance in the standardized neuropsychological tests was eight times significantly increased in the post- compared to the pre-test. Whereas the controls showed five times significant performance improvements when comparing post- and pre-test (four out of eleven controls).

Both groups showed an increased performance in the Benton Test after VR training compared to the pre-test. The Benton Test assesses visual perception and visual memory [27]. Patients showed a Benton score of 10.18 points before VR training, which is associated with impaired visual perception and memory [27]. After VR training, patients with spatial disorientation reached an average score of 13.36 points, which is associated with normal visual perception and visual memory performance. Hence, after navigation training in VR the visual perception and visual memory of patients with spatial deficits improved from a neurologic impaired level to a normal level. The healthy control group showed a normal visual perception and visual memory performance (12.64 points in the Benton Test) already before VR training. Nevertheless, they could increase their performance up to 14.73 points after VR training. The maximum score of the Benton Test is 15 points. Hence, the control group showed a nearly perfect performance in the Benton Test after five VR training sessions. This result is in line with the findings of Caglio et al. (2012) who demonstrated that spatial navigation training in VR can improve visual-spatial memory learning in a patient with traumatic brain injury through the exploration of a new and complex virtual environment. In the present study, the improvement in quality of spatial memory can be directly related to the need to memorize different routes in a complex VR environment.

In the subtest spatial imagination of the Achievement Measure System 50+ (LPS 50+), the ANOVA revealed a significant main effect of time, which indicates that controls as well as patients improved their performance in the LPS 50+ after VR training. However, the subsequent t-test showed that only the patient group showed a significant improved performance after five VR training sessions compared to the pre-test. Before the VR training sessions, the patient group showed a mean LPS 50+ score (T-score) of 44.73, which was more than a half standard deviation below the average of the normal distribution (Mean = 50.00; *SD* = 10.00) [38]. After VR training, neurologic patients could improve their performance in the LPS 50+ with a resulting mean T-score of 50.00. Hence, the ability to imagine spatial objects and to mentally transform them increased in patients with spatial disorientation after VR training. The LPS 50+ scores of neurologic healthy elderly were above the average of the normal distribution in the pre- (58.09) and post-test (59.82). The most prominent ability common to both LPS 50+ and VR training is mental rotation and representation. The ability to construct a mental map of the route might have been recruited in VR training, since participants had to compute their routes forwards as well as backwards, and generalized to the performance in the LPS 50+.

In the Visual Pursuit Test (LVT) [30], which is one of the two computer based spatial tests, patients with spatial deficits answered faster after VR training than before. The significant interaction time x group observed in the median time score of the LVT indicates that patients benefited more from VR training than matched controls. Hence, in the patient group the reaction time during visual orientation performance for simple structures in a complex environment decreased after VR training. The healthy control group already answered very fast during the pre-test (4.35 s), therefore, a decrease in reaction time was hardly possible. No changes in overall performance (score) of the LVT could be found between the pre- and post-test, probably because participants had to find the right answer while being placed under time pressure. If the participants did not give an answer in a predefined time window, the answer did not count any more even when it was correct. Hence, many correct answers were not counted because the participants answered too slowly. In contrast, for the median time of correct answers the reaction times of all correct answers were taken into account, even if they were not given in the predefined time window. Therefore, the median time of correct answers might be a more appropriate measure when testing patients with brain damage, who generally need more time to process different tasks, than the absolute score of the LVT. In summary, more general aspects of visual selective attention measured by the LVT responded positively to VR training. One natural reason for that is the need to rapidly update visually presented information in both LVT and VR tasks.

In the computer-based Corsi Block-Tapping Test (CBTT) [32] neither the healthy control group nor the patient group showed a significant improvement in performance between the pre- and post-test. Both groups showed slightly higher scores in the post- than in the pre-test. Nevertheless, these marginal performance improvements did not reach significance. The CBTT primarily assesses visual short-term memory capacity [32], which might not have improved due to the spatial way-finding training in VR [15].

Due to motor impairments, not all neurologic patients with spatial deficits were able to complete the computer-based tests LVT and CBTT. Hence, these computer based spatial tests are not suitable for all patients with brain damage.

In summary, these results allow to generalize the obtained VR training outcomes to more general aspects of spatial cognition. The spatial way-finding training in a specific virtual environment could enhance general spatial abilities assessed with specific neuropsychological tests in neurologic patients with spatial orientation problems as well as in healthy controls. Hence, the VR-based verbally guided spatial navigation training used in the present study is associated with improvements in some general aspects of spatial cognition.

However, based on our results we cannot conclude that neurologic patients with spatial orientation problems show an improved spatial orientation performance in the real world after VR training, such as a more autonomous navigation through the real hospital area, their own home town, or the real world district of Graz, which was used as VR training environment in the present study. Several prior studies investigated the transfer of spatial knowledge from a virtual to a corresponding real environment and provided evidence that VR based spatial learning transfers to improved performance in the real world [16,19,39-44].

Furthermore, a high priority for future work is to examine changes in general spatial abilities in a control group, defined by a neurologic patient group with spatial orientation problems that undergoes no VR navigation training. With such a control group one could prove if improvements in general aspects of spatial abilities are only caused by the VR training or if the effects are caused by general time on task effects independent of the intermediate spatial training. Additionally, follow-up measurements would be necessary to examine possible long-term training effects.

In both groups, performance in the VR route finding task was positively correlated with performance in standardized neuropsychological tests. This indicates that the spatial navigation task in the virtual environment is a useful tool to assess spatial abilities and that it is largely valid. These correlation results are in accordance with the findings of Kalová et al. (2005) and Cushman et al. (2008) who could show that VR is a valid assessment method of spatial skills [10-12]. Morganti et al. (2007) also compared spatial performance in VR with performances in standardized neuropsychological tests, such as the Digit and Corsi’s span test, the Progressive Raven’s Matrixes test, the Trial Making test, the Rey’s complex figure test, or the Benton’s line orientation test. Therefore, they tested patients with brain damage and healthy matched control participants. Brain damaged patients performed worse than healthy controls in all tasks. Hence, the patients’ performance in the VR task was congruent with their neuropsychological evaluation [13]. Moffat et al. (2001) also found a positive relationship between spatial navigation performance in a virtual maze and the performance in standardized neuropsychological tests, such as the Benton Visual Retention Test, in healthy elderly [45].

In the present study, all neurologic patients had spatial orientation problems, although they showed various brain lesion sites. Studies examining neuroanatomical correlates of TD showed that TD is associated with lesions in both hemispheres of the brain. Hence, TD can occur after brain lesions at different sites [46]. Carelli et al. (2011) examined spatial abilities of patients with brain damage in VR, too. These patients also showed differences in lesion sites [47]. Carelli et al. (2011) could not find any relationship between lesion site and spatial performance neither in the VR task nor in standardized neuropsychological tests such as the Corsi Block-Tapping Test, the Benton’s line orientation test for line orientation judgement, or the Trial Making test to assess divided attention [47]. Compared to a healthy matched control group, patients with brain damage showed an impaired VR task performance, such as in the current study [47]. Hence, different spatial deficits associated with different brain lesions might contribute to influence the performance in VR spatial tasks. Furthermore, when referring to the results of the single-case analysis, no systematical differences between performance improvements in standardized neuropsychological tests and lesion location or time since onset can be seen (see Table 1 and Table 4). Nevertheless, we cannot exclude that patients with different brain lesions who show distinct spatial impairments demonstrate different learning effects or the absence thereof in the present VR navigation training. Further studies are needed to investigate the influence of brain lesion site on VR navigation performance in more detail.

## Conclusion

In summary, the current study provides evidence that our VR-based verbally-guided passive navigation training program can enhance general aspects of spatial abilities in neurologic patients with spatial orientation problems as well as in healthy controls. Patients with spatial deficits and matched controls showed an improved performance in standardized neuropsychological tests assessing general spatial abilities after only five VR training sessions compared to the pre-test. Prior VR-based spatial training studies focused on improvements in specific spatial performances in particular environments that also were used as training environments. For instance, Lloyd et al. (2009) trained and tested participants’ route finding ability in the same virtual town. General spatial abilities of the patients participating in their study were not assessed [21]. Wilson et al. (1996) and Brooks et al. (1999) performed a spatial training in a virtual environment and tested the acquired spatial knowledge in the corresponding real world unit. Possible changes in general spatial abilities due to VR training were not examined either [16,19]. Hence, this is the first study in which the influence of a specific spatial training in VR on general spatial abilities was investigated.

To date, rehabilitation of spatial orientation ability after brain damage is generally a part of common therapy sessions at the rehabilitation hospital and there is no explicit training of navigation skills or general spatial abilities in use [1]. Therefore, spatial way-finding training in VR might provide a new and ecologically valid rehabilitation method of spatial deficits. Our results indicate that VR is potentially useful in the rehabilitation of spatial deficits associated with TD.

## Appendix I

Calculation of weighted total score: Per training session maximal four routes could be learned. If a previously learned route was recalled correctly without any error in the first run, the participant got 24 points for this route and the learning phase of the next route started. Hence, if all four routes were performed without any errors, the participant could reach maximum 96 points per training session. If no route was performed correctly, the participant got 0 points. Each route contained three junctions where the participants had to make a decision on the direction. The participants had to recall this route from the starting to the endpoint and backwards after the learning phase. Hence, per run maximum 6 errors could be made. If a participant made an error in the first run (×_1_ = number of errors in first run), but completed the second run error-free, the following formula was used to calculate the points for the actual route: ((24-×_1_*4)*0.25 + 18). If a participant made an error in the second run too (×_2_ = number of errors in second run), but completed the third run error-free, the following formula was used to calculate the points for the actual route: ((24-×_1_*4)*0.25 + (18-×_2_*3)*0.15 + 12). If a participant also made an error in the third run (×_3_ = number of errors in third run), but completed the fourth run error-free, the following formula was used to calculate the points for the actual route: ((24-×_1_*4)*0.25 + (18-×_2_*3)*0.15 + (12-×_3_)*0.1 + 6). If a participant made an error in the fourth run (×_4_ = number of errors in fourth run), the following formula was used to calculate the points for the actual route: ((24-×_1_*4)*0.25 + (18-×_2_*3)*0.15 + (12-×_3_)*0.1 + 6 - ×_4_). Maximum 4 runs per route were possible. The resulting weighted total score was the sum of the reached points of all learned routes per training session.

## Competing interests

The authors declare that they have no competing interests.

## Authors’ contributions

SEK conceived of the study and made substantial contributions to conception and design of the study, performed the statistical analysis, made the analysis and interpretation of data, and drafted the manuscript. GW has been involved in the interpretation of data, drafting the manuscript and revising it critically for important intellectual content. DH participated in the design of the study, performed statistical analysis and collected the data. WK & MK participated in the selection and medical care of the neurologic patients, coordination, data collection and interpretation. CN participated in the design of the study and interpretation of data, revised the manuscript critically for important intellectual content. All authors read and approved the final manuscript.
